# Multimarker Analysis of Serially Measured GDF-15, NT-proBNP, ST2, GAL-3, cTnI, Creatinine, and Prognosis in Acute Heart Failure

**DOI:** 10.1161/CIRCHEARTFAILURE.122.009526

**Published:** 2022-11-21

**Authors:** Muhammed T. Gürgöze, Laura C. van Vark, Sara J. Baart, Isabella Kardys, K. Martijn Akkerhuis, Olivier C. Manintveld, Douwe Postmus, Hans L. Hillege, Ivonne Lesman-Leegte, Folkert W. Asselbergs, Hans-Peter Brunner-la-Rocca, Ewout J. van den Bos, Joke G. Orsel, Stijn P.J. de Ridder, Yigal M. Pinto, Eric Boersma

**Affiliations:** Department of Cardiology, Thorax Center, Erasmus MC, University Medical Center Rotterdam, the Netherlands (MT.G., L.C.v.V., I.K., K.M.A., O.C.M., E.B.).; Department of Biostatistics, Erasmus MC, University Medical Center Rotterdam, the Netherlands (S.J.B.).; Department of Epidemiology (D.P., H.L.H.), University Medical Center Groningen, University of Groningen, the Netherlands; Department of Cardiology (H.L.H.), University Medical Center Groningen, University of Groningen, the Netherlands; Department of General Practice and Elderly Care Medicine (I.L.-L.), University Medical Center Groningen, University of Groningen, the Netherlands; University Medical Center Groningen, University of Groningen, the Netherlands (H.L.H.).; Department of Cardiology, Division of Heart and Lungs, University Medical Center Utrecht, Utrecht University, the Netherlands (F.W.A.).; Institute of Cardiovascular Science, Faculty of Population Health Sciences (F.W.A.), University College London, United Kingdom.; Health Data Research UK and Institute of Health Informatics (F.W.A.), University College London, United Kingdom.; Department of Cardiology, Maastricht University Medical Center, the Netherlands (H.-P.B.-l.-R.).; Department of Cardiology, Albert Schweitzer Hospital, Dordrecht, the Netherlands (E.J.v.d.B.).; Philips Healthcare, Eindhoven, the Netherlands (J.G.O.).; Department of Cardiology, St. Anna Hospital, Geldrop, the Netherlands (S.P.J.d.R.).; Department of Experimental Cardiology, Academic Medical Center, Amsterdam, the Netherlands (Y.M.P.).

**Keywords:** biomarkers, growth differentiation factor 15, heart failure, prognosis

## Abstract

Studies on serially measured GDF-15 (growth differentiation factor 15) in acute heart failure (HF) are limited. Moreover, several pathophysiological pathways contribute to HF. Therefore, we aimed to explore the (additional) prognostic value of serially measured GDF-15 using a multi-marker approach to more accurately predict HF risk.

**Methods:**

TRIUMPH (Translational Initiative on Unique and Novel Strategies for Management of Patients With Heart Failure) is a prospective cohort of 496 patients with acute HF who were enrolled in 14 hospitals in the Netherlands between 2009 and 2014. Blood sampling was scheduled at 7 moments during 1-year follow-up. GDF-15, NT-proBNP (N-terminal pro-B-type natriuretic peptide), ST2 (suppression of tumorigenicity 2), galectin-3, troponin I, and creatinine were measured in a central laboratory. We associated repeated measurements of these biomarkers with the composite primary end point of all-cause mortality and HF rehospitalization, using multivariable joint modeling.

**Results:**

Median age was 74 years, and 37% were women. Median baseline GDF-15 was 4632 pg/mL. The primary end point was reached in 188 (40%) patients. The average estimated GDF-15 level increased weeks before the primary end point was reached. The hazard ratio per 1 SD difference in log-GDF-15 was 2.14 (95% CI, 1.78–2.57) unadjusted, 1.96 (1.49–2.53) after adjustment for clinical confounders and 1.44 (1.05–1.91) when jointly modeled with all biomarkers. The adjusted HRs for NT-proBNP were 2.38 (1.78–3.33) and 1.52 (1.15–2.08), respectively. The multimarker model combining GDF-15, NT-proBNP, and troponin I provided a favorable risk discrimination (area under the curve=0.785).

**Conclusions:**

Sequentially measured GDF-15 independently and dynamically predicts risk of adverse outcomes during 1-year follow-up after index admission for acute HF. NT-proBNP remains a robust predictor among potential candidates. Multiple biomarkers should be considered for stratification in clinical practice.

**Registration:**

URL: https://www.trialregister.nl/trial/1783; Unique Identifier: NTR1893. (The trial can be found temporarily at https://trialsearch.who.int/Trial2.aspx?TrialID=NTR1893.)

**What is New?**

- Increase in GDF-15 (growth differentiation factor 15) level is strongly associated with an increased composite risk of all-cause mortality and heart failure hospitalization after admission for acute heart failure, independent of multiple biomarkers including NT-proBNP (N-terminal pro-B-type natriuretic peptide).
- Repeated measurements better reflect the dynamic pattern of biomarkers and take into account the natural disease progression compared with a single, baseline measurement.
- A multimarker panel of GDF-15, NT-proBNP, and troponin I has a stronger relation with the incidence of adverse outcomes during follow-up than a single-marker panel.

**What are the Clinical Implications?**

- A combination of multiple, serially measured (novel and established) biomarkers could improve risk stratification on top of existing risk prediction tools and further facilitate clinical decision-making.
- Frequent measurement of biomarkers during outpatient follow-up could provide an actionable window to anticipate and potentially prevent adverse events such as readmission, warranting further research into biomarker-guided management of heart failure.

**See Editorial by Alzate et al**

Heart failure (HF) increasingly burdens health care costs^1^ due to high mortality rates and frequent hospitalization despite evidence-based treatment according to current guidelines.^2^ In the context of reducing this growing burden, serum biomarkers, which reflect underlying biological processes, are becoming increasingly popular for risk stratification and treatment guidance. The most well-known and extensively studied biomarker in HF is NT-proBNP (N-terminal pro-B-type natriuretic peptide), which has been shown to provide incremental prognostic value to known clinical confounders. However, HF is a syndrome with a broad pathophysiological basis, and there is still need for novel circulating biomarkers that are expressed downstream several relevant molecular pathways.^3^ Recent examples of such novel HF biomarkers include ST2 (suppression of tumorigenicity 2)^4^ and galectin-3,^5^ which we have previously investigated and shown to provide additional information to that conferred by NT-proBNP. Despite this evidence, these markers have not yet been adopted in the guidelines or routine clinical care.

A promising upcoming HF biomarker, which we have not previously investigated, is GDF-15 (growth differentiation factor 15). GDF-15 is a member of the transforming growth factor beta cytokine superfamily that is expressed in inflammatory state, under oxidative stress and reflects cardiac remodeling.^6,7^ A meta-analysis of 8 clinical studies in patients with HF showed that elevated levels of GDF-15 were associated with increased mortality.^8^ However, these studies relied on a single, baseline measurement of GDF-15, which fails to take into account disease progression and the dynamic pattern of biomarkers during follow-up. Studies on the longitudinal evolution of GDF-15 (≥3 measurements) and its relation with HF prognosis are limited^9–12^ and even more so in patients with acute HF.^10^ Furthermore, a multimarker approach might be necessary to account for the heterogeneity in pathophysiology and has been insufficiently applied in this context.^9,10^ Thus, the full potency of serially measured GDF-15 remains unclear.

The current article describes our findings with respect to repeated measurements of several biomarkers, which we studied as prognostic markers for relevant clinical outcomes, with particular interest in the additional prognostic value of GDF-15 as part of a multimarker approach including NT-proBNP, ST2, galectin-3, troponin I, and creatinine. To this end, we used our TRIUMPH study (Translational Initiative on Unique and Novel Strategies for Management of Patients With HF), which was typically designed for this purpose; to identify and validate the prognostic value of temporal patterns of potentially relevant biomarkers in patients with acute HF.^4,5^

## Methods

### Data Integrity and Sharing

The corresponding author had full access to all the data in the study and takes responsibility for its integrity and the data analysis. The data that support the findings of this study are available from the corresponding author upon reasonable request.

### Study Design and Procedures

Full details of the TRIUMPH study have been published before^4,5^ and are briefly mentioned here. TRIUMPH was a prospective, observational study conducted in 14 hospitals in the Netherlands between September 2009 and December 2013, enrolling patients admitted with acute HF. Patients were eligible if they were ≥18 years and hospitalized with a diagnosis of acute HF, either newly diagnosed or as an exacerbation of known, chronic HF. During hospitalization blood samples were collected at day 1 (admission), day 2 to 4, and on the day of discharge. Hereafter, blood samples were collected during regular outpatient follow-up visits at 2 to 4 weeks, 3, 6, and 9 to 12 months. The baseline blood sample was defined as the first measurement obtained within 48 hours after inclusion. HF status was assessed at each visit using New York Heart Association classification. Medication use was determined at discharge. Patients underwent physical examination, venipuncture, and imaging (including echocardiography), and all relevant variables were systematically measured during the scheduled moments described above. Follow-up was up to a maximum of 400 days after index admission to allow assessment of biomarker changes near the end point. The primary end point (PE) was the composite of all-cause mortality and HF hospitalization. The secondary outcome was all-cause mortality. An event adjudication committee, blinded to biomarker information, reviewed and adjudicated the study end points.

This study complies with the Declaration of Helsinki and was approved by the METC Erasmus MC institutional review board (MEC 2009-053) as well as the review boards at all other participating centers. It has been registered in the national trial register (NTR1893). All patients provided written informed consent before study procedures. The procedures followed were according to institutional guidelines. Patients received care as usual by the treating physician according to the prevailing HF guidelines at the time.^13^ The treating physician was blinded to study-specific biomarker data, which was measured after study completion.

### Blood Samples and Biomarker Measurements

Nonfasting blood samples were drawn by means of venipuncture and transported to the clinical chemistry laboratory of each participating center for further processing according to a standardized protocol. Samples were centrifuged at 1700 G/relative centrifugal force, after which heparin plasma and blood serum were separated. All blood aliquots were stored at a temperature of −80 °C within 2 hours after venipuncture.

All samples were measured in a single batch analysis of GDF-15, NT-proBNP, ST2, galectin-3, troponin I, and creatinine levels at a central laboratory. GDF-15 levels were determined in serum by the Cobas-e system using the Roche Diagnostics GDF-15 electro-chemiluminescent sandwich immunoassay (Elecsys GDF-15). NT-proBNP levels were determined in heparin plasma by using the Elecsys NT-proBNP electro-chemiluminescent sandwich immunoassay on a Cobas 8000 analyzer (Roche Diagnostics Ltd, Rotkreuz, Switzerland). ST2 levels were determined in serum using a quantitative sandwich monoclonal ELISA (Presage ST2 Assay; Critical Diagnostics Inc, San Diego, CA). Galectin-3 levels were determined in serum using the BGM galectin-3 Test (BG Medicine Inc, Waltham, MA). Troponin I levels were determined in heparin plasma on an Access 2 immunoassay system using the Access AccuTni assay procedure (Beckman Coulter Inc, Fullerton, CA). creatinine levels were determined in heparin plasma on the Cobas 8000 analyzer.

Analysts were blinded to patient characteristics and study end points.

### Statistical Analysis

All continuous variables were non-normally distributed, as assessed by visual examination of histograms and Q-Q plots. Continuous variables are therefore presented as median and interquartile range (IQR), and differences in continuous variables between baseline GDF-15 quartiles were evaluated using the Jonckheere-Terpstra trend test. Categorical variables are presented as counts and percentages, and differences in categorical variables between baseline GDF-15 quartiles were evaluated with χ^2^ trend tests using the Cochran-Armitage extension or the linear-by-linear association according to Mantel-Haenszel, as appropriate. The biomarkers were log-transformed and the correlation between biomarkers was calculated using Spearman correlation analyses. The log-transformed biomarkers were then standardized, and their Z-scores were used for longitudinal analyses.

The association between baseline biomarker measurement and study end points was assessed using Cox proportional hazards (PH) regression models. The PH assumption was evaluated based on the scaled Schoenfeld residuals. The association between repeated biomarker measurements and study end points was assessed using joint models, which combine a linear mixed effects model for the longitudinal evolution of the biomarker with a time-to-event model that relates the serially measured biomarker levels to the incidence of the end points.^14^

We ran the following models:

1. Univariable or unadjusted.
2. Adjusted for age, sex, systolic blood pressure, diabetes, left ventricular ejection fraction, previous HF hospitalization within last 6 months, ischemic HF etiology, body mass index, estimated glomerular filtration rate^15^ (except in model with creatinine; clinical model).
3. Adjusted for clinical variables and NT-proBNP.
4. Adjusted for clinical variables and NT-proBNP plus an additional biomarker.
5. Only adjusted for all biomarkers (biomarker model).

The selection of potential confounders is based on previous analyses of TRIUMPH and represents some of the common variables also used in risk assessment tools like Meta-Analysis Global Group in Chronic HF^16^ and Barcelona (BCN) Bio-HF calculator^17^. Both the linear mixed effects and Cox PH regression submodels were adjusted for the same variables. We used cubic splines with knots set at 1 week and 1 month after index admission for the linear mixed effects submodel, based on clinical data and biomarker evolution. The results of the models are presented as hazard ratios (HRs) per 1 SD difference of the biomarker level (on the log-scale) with 95% CIs. Measures of discrimination (C-index and area under the curve) are also presented for each of the models. The area under the curve was based on the “aucJM” function with measurements up to 7 days used to predict outcomes up to 30 days for short-term and similarly 30 to 400 days for long-term. Data on covariates were complete in at least 92%, except for left ventricular ejection fraction with 78% completeness. Missing data in covariates were addressed by means of single imputation using the multivariate imputation by chained equations function.

For all tests, a *P*<0.05 was considered statistically significant. Data were analyzed using SPSS Statistics for Windows, version 25 (IBM Corp, Armonk, NY) for data preparation and descriptive analyses. R Statistical Software version 3.6.3 (Vienna, Austria) was used for the main analyses; Cox regression analysis using the “survival” package, joint modelling with “mvJMBayes” function within the “JMBayes” package.^14^

## Results

### Baseline Characteristics

The TRIUMPH cohort study enrolled 496 patients. However, 3 patients withdrew informed consent, whereas 18 patients were withdrawn from analysis due to a lack of evidence of sustained left ventricular dysfunction. Therefore, the analysis set included 475 patients; baseline characteristics are presented in Table 1. Median age was 74 years (IQR, 65–81), and 37% were women. The median left ventricular ejection fraction was 30% (IQR, 21–41), and most patients (83%) had HF with reduced ejection fraction according to the prevailing HF guidelines at the time^13^ whereas this was 69% according to the updated guidelines.^2^ More than half (55%) of the patients were in New York Heart Association class III. Median baseline levels of GDF-15, NT-proBNP, ST2, galectin-3, troponin I, and creatinine were 4632 pg/mL (IQR, 2859–7399), 4152 pg/mL (IQR, 2089–9387), 72 ng/mL (IQR, 47–103), 24 ng/mL (IQR, 18–34), 46 ng/mL (IQR, 24–99), and 126 µmol/L (IQR, 100–164), respectively.

**Table 1.**
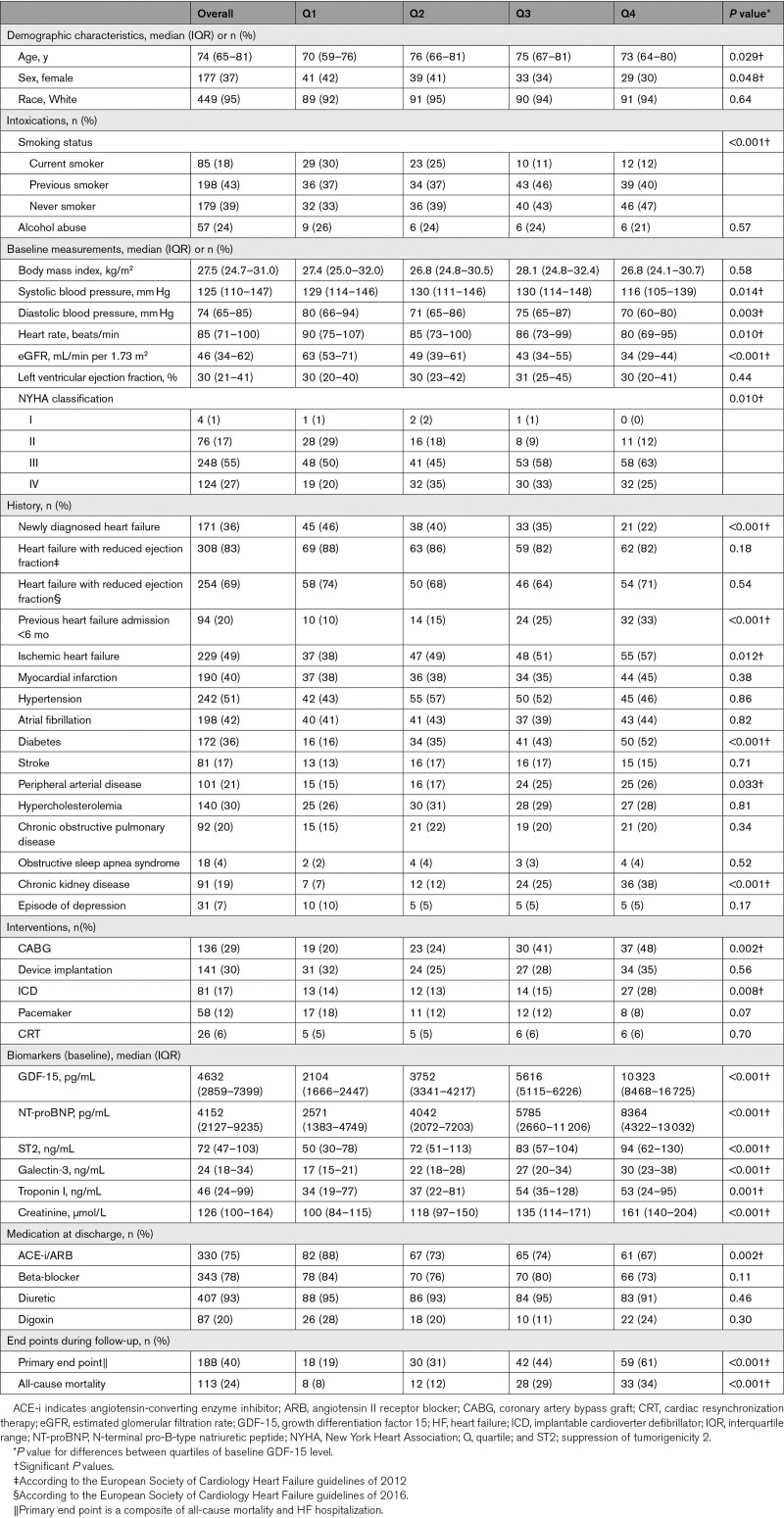
Characteristics and Study End Points Overall (n=475) and for Quartiles of Baseline GDF-15 Level (n=386)

Table 1 also shows the characteristics according to quartiles of baseline GDF-15. Kidney function was significantly worse in the highest quartile compared with the lowest (34 versus 63 mL/min per 1.73 m^2^) and as expected, the prevalence of chronic kidney disease was higher (38% versus 7%; *P*<0.001). Similarly, more patients in the higher quartiles had undergone previous HF hospitalization in the last 6 months, had ischemic HF etiology and diabetes. Importantly, the opposite was true for patients with new-onset HF as nearly half of the patients in the lowest quartile of GDF-15 had new-onset HF compared with the highest (46% versus 22%; *P*<0.001). Most patients used diuretics (93%), beta-blocker (78%), or ACE-i/ARB (75%) and use of the latter was significantly lower in the highest quartile (67% versus 88%; *P*=0.002). Across the board, baseline biomarker levels were significantly higher in the highest quartile compared with the lowest.

### Study End Points

The PE was reached in 188 (40%) of the patients during a median follow-up of 325 days (IQR, 85–401; Table 1). A total of 113 patients (24%) died of any cause (68% cardiovascular) during follow-up. In the highest GDF-15 quartile, 61% of the patients reached the PE, while this was 19% in the lowest (*P* <0.001). A similar pattern was observed for all-cause mortality.

### Correlations Between Biomarkers

**Figure 1.**
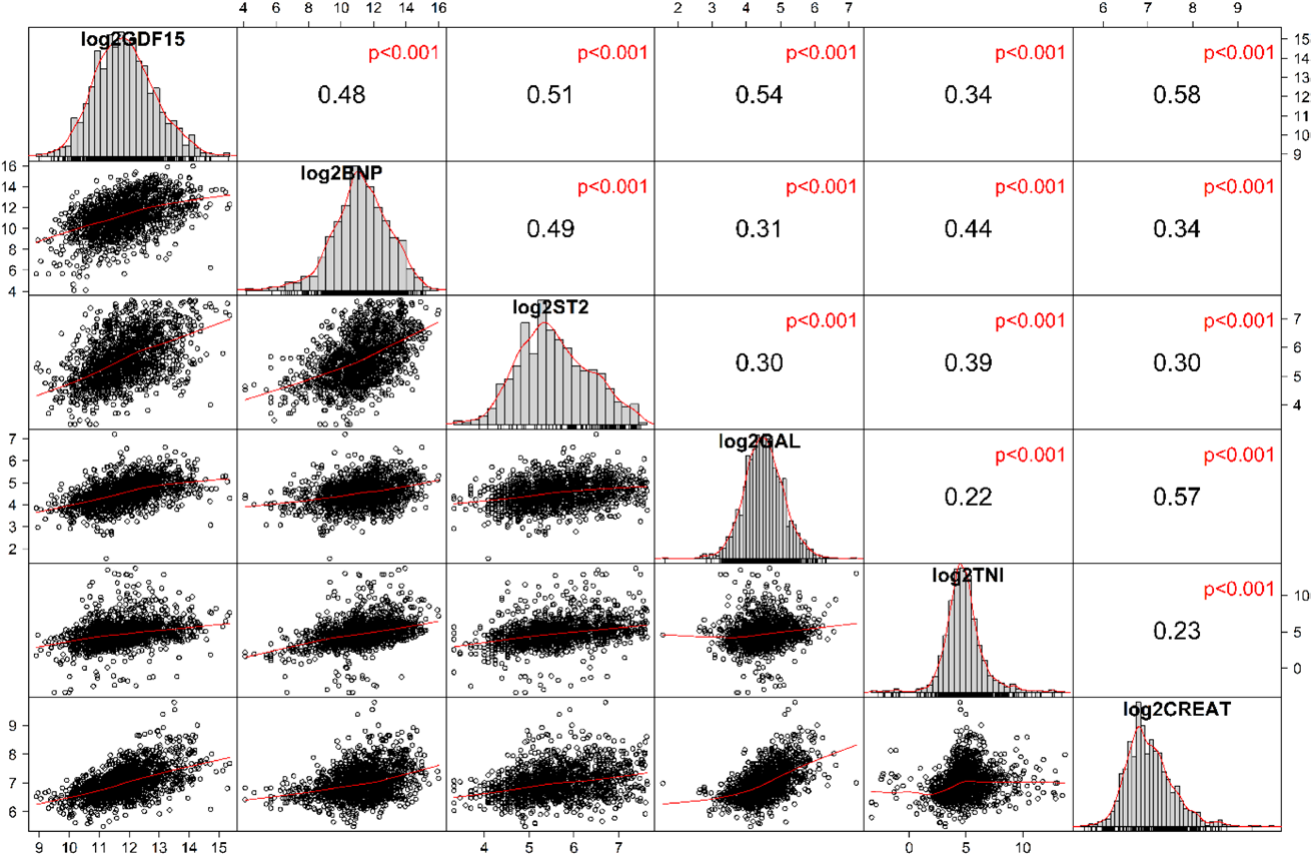
**Correlation plot of all biomarkers.** Correlation plot showing the associations between biomarkers based on the log-transformed values. Diagonally depicted are the individual distributions of the biomarkers. Below the diagonal are presented the scatter plots of the correlations between biomarkers, and above the diagonal are presented the corresponding Spearman correlation coefficients with *P* values. BNP indicates N-terminal pro-B-type natriuretic peptide; CREAT, creatinine; GAL, galectin-3; GDF15, growth differentiation factor 15; log2, logarithm to the base 2; ST2, suppression of tumorigenicity 2; and TNI, troponin I.

The correlation between all 6 biomarkers is shown in Figure 1. All biomarkers showed a near normal distribution on the log-scale. There was statistically significant correlation between all biomarkers on the log-scale. The correlation was strongest between GDF-15 and creatinine. The pairs GDF-15 and galectin-3, GDF-15 and ST2, as well as creatinine and galectin-3 also showed an association. Based on the coefficients, these relationships were moderate at best.

### Baseline Measurement and Prognosis

The PH assumption of the Cox PH regression analyses appeared satisfied. Univariable HR (model 1) per 1 SD difference of GDF-15 for the PE was 1.67 ([95% CI, 1.39–2.02]; *P*<0.001; Table 2). According to the clinical model (2) the adjusted HR was 1.42 ([95% CI, 1.12–1.80]; *P*=0.003) and additionally adjusted for NT-proBNP (model 3) it was 1.28 ([95% CI, 1.00–1.63]; *P*=0.05). Jointly modeled with NT-proBNP, ST2, galectin-3, troponin I and creatinine (biomarker model 5) the HR was 1.41 ([95% CI, 1.11–1.80]; *P*=0.005). The multimarker model combining GDF-15, NT-proBNP, and troponin I provided a favorable risk discrimination (C-index=0.722) in comparison to a single-marker model. A similar pattern was observed for all-cause mortality (Table 3). Notably, the associations were stronger for the mortality end point than the composite PE. Overall, NT-proBNP was the strongest predictor and independently associated with the end points in all models followed by GDF-15 and ST2 as strong candidates. Furthermore, troponin I had significant incremental prognostic value for the mortality end point but not for the PE in combination model 4.

**Table 2.**
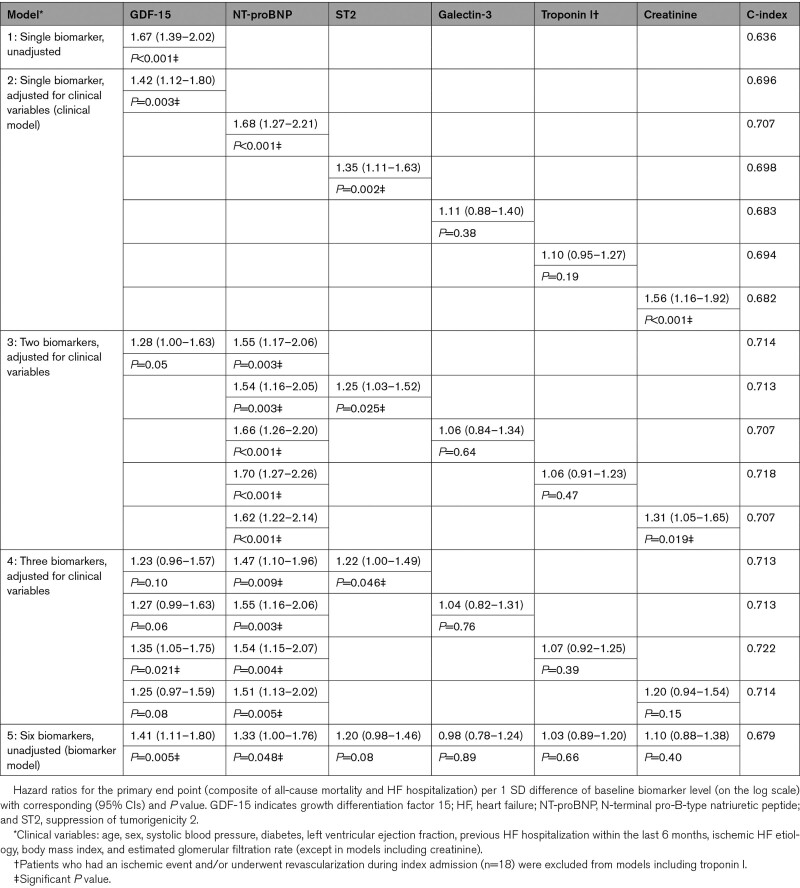
Association of Baseline Measurement of Biomarkers With the Primary End Point

**Table 3.**
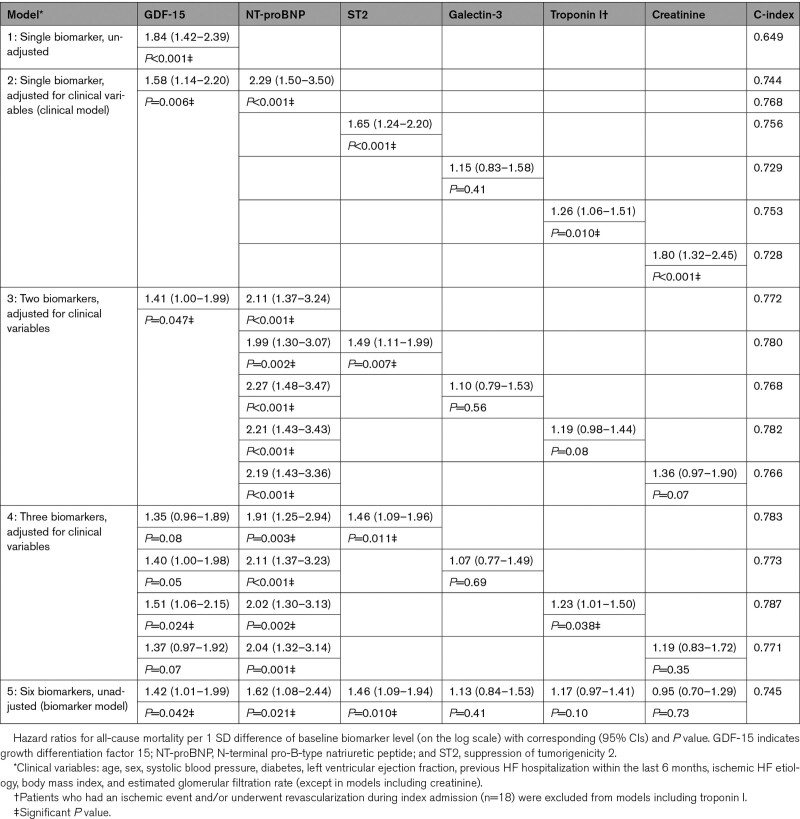
Association of Baseline Measurement of Biomarkers With All-Cause Mortality

### Repeated Measurements and Prognosis

**Figure 2.**
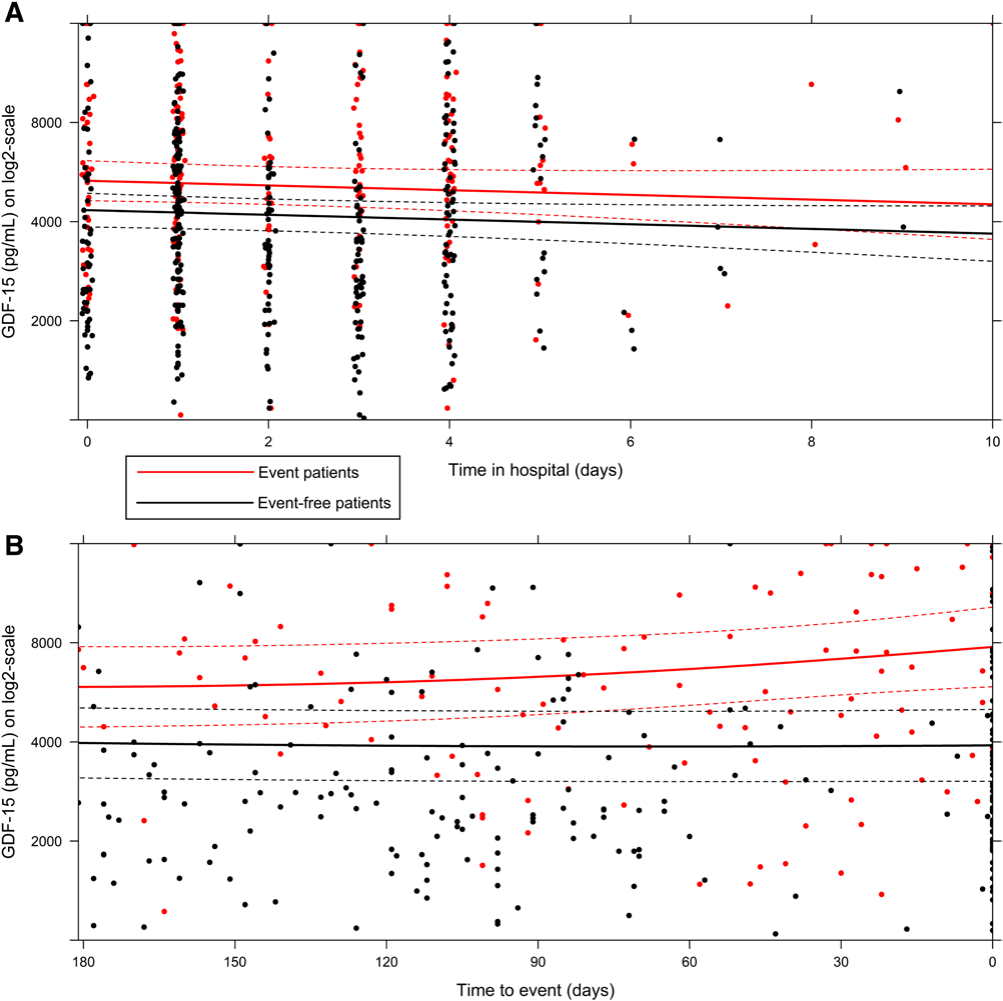
**Temporal pattern of average estimated GDF-15 (growth differentiation factor 15) level during index admission and follow-up in patients with and without the primary end point. A**, Longitudinal evolution of average estimated GDF-15 level during index admission. **B**, Longitudinal evolution of average estimated GDF-15 level during follow-up until the event or end of follow-up. Patients who reached the primary end point during follow-up are shown in red and those who did not in black. The *y* axis is on a logarithmic scale with the raw values shown. Solid bold lines represent mean values; dashed lines represent the corresponding 95% CI. Dots represent individual measurements. The average GDF-15 level is based on a linear mixed effects model adjusted for age, sex, systolic blood pressure, diabetes, left ventricular ejection fraction, previous heart failure (HF) hospitalization within last 6 months, ischemic HF etiology, body mass index, estimated glomerular filtration rate, and NT-proBNP (N-terminal pro-B-type natriuretic peptide). log2 indicates logarithm to the base 2. Adapted from Abstracts of the Heart Failure 2021 and the World Congress on Acute Heart Failure^33^ with permission. Copyright ©2021, European Society of Cardiology.

The average number of repeated measurements per patient during follow-up was 3.6 for GDF-15, 3.9 for ST2 and 4.1 for NT-proBNP, galectin-3, troponin I, and creatinine. Figure 2 shows the longitudinal evolution of the average estimated GDF-15 level during index admission and during follow-up. The *x* axis is reversed in the bottom graph showing the period leading up to the PE or end of follow-up. The average estimated GDF-15 level was higher in patients who reached the PE versus those who did not and decreased during index admission in both groups following treatment for decompensation. The average estimated GDF-15 level increased weeks before the PE was reached while levels remained stable in patients who did not reach the PE.

The univariable HR per 1 SD difference of GDF-15 for the PE was 2.14 ([95% CI, 1.78–2.57]; *P*<0.001; Table 4). According to the clinical model, the HR was 1.96 ([95% CI, 1.49–2.53]; *P*<0.001) and additionally adjusted for NT-proBNP it remained statistically significant. In the biomarker model, the HR was 1.44 ([95% CI, 1.05–1.91]; *P*=0.022). The multimarker model combining GDF-15, NT-proBNP, and troponin I provided a favorable risk discrimination (area under the curve=0.785) in comparison to a single-marker model. A largely similar pattern with stronger associations was observed for the mortality end point (Table 5). NT-proBNP was again the strongest predictor. Unlike the baseline analysis, serially measured troponin I had significant incremental prognostic value for the PE in the biomarker model and the association was largely driven by all-cause mortality. In both analyses, the additional prognostic value of galectin-3 and creatinine was mostly limited.

**Table 4.**
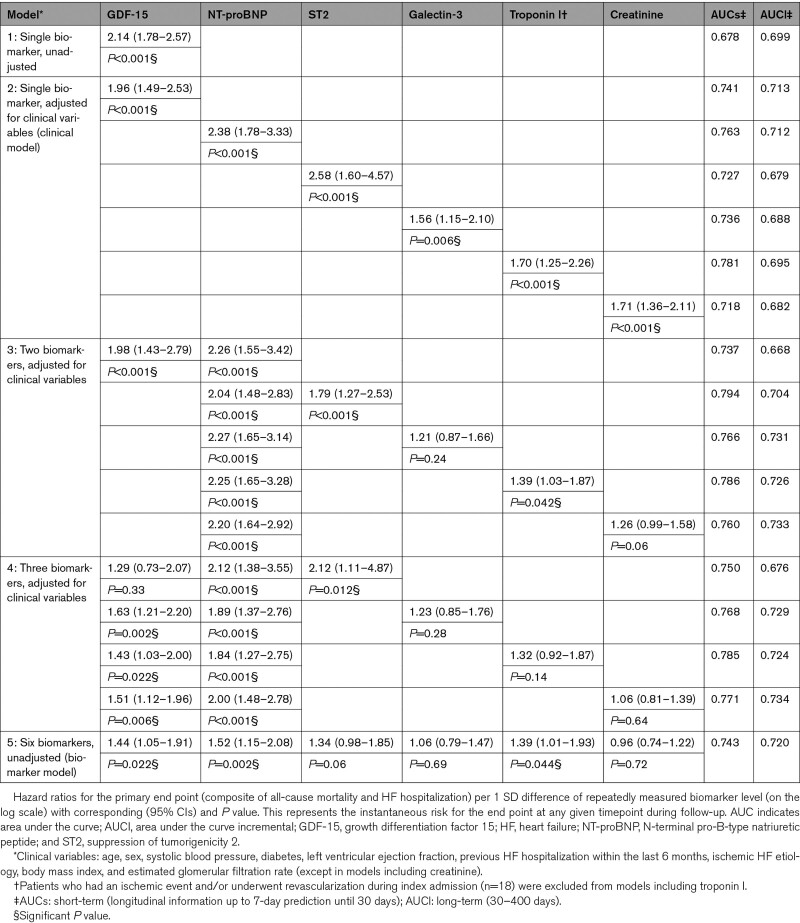
Association of Repeated Measurements of Biomarkers With the Primary End Point

**Table 5.**
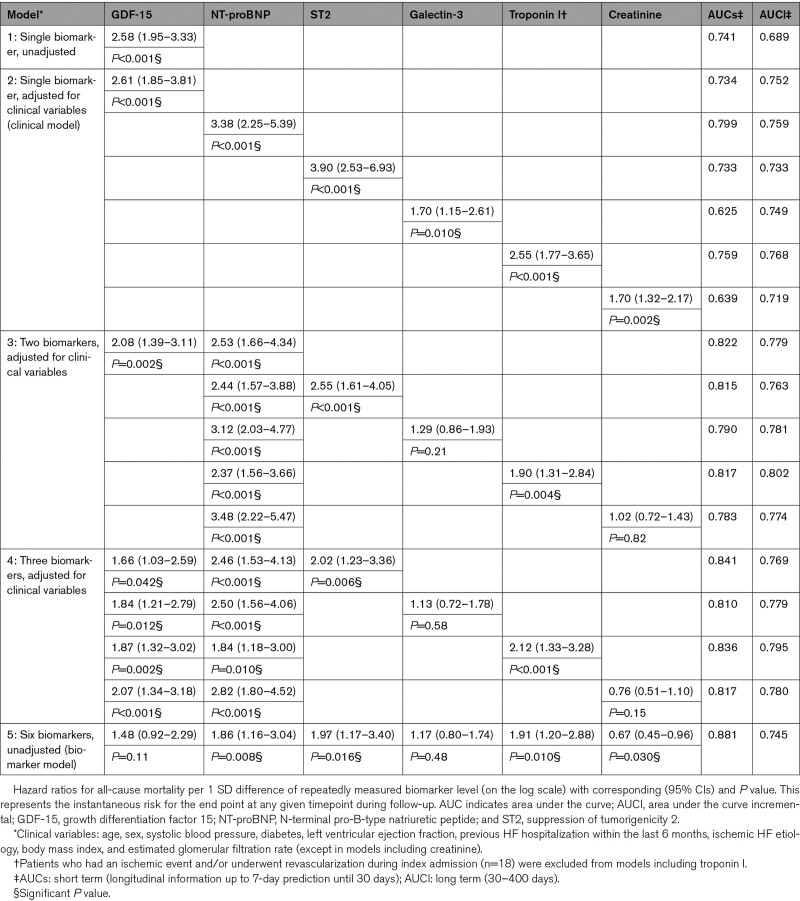
Association of Repeated Measurements of Biomarkers With All-Cause Mortality

## Discussion

In this study of 475 patients with acute HF, we show that serially measured GDF-15 dynamically predicts the composite risk of all-cause mortality or HF rehospitalization during 1-year follow-up independent of several other serially measured biomarkers including NT-proBNP. Moreover, the multimarker model combining GDF-15, NT-proBNP, and troponin I provides a favorable risk discrimination. Troponin I provides incremental prognostic value mainly for all-cause mortality while galectin-3 and creatinine have limited additional value for both end points. Overall, NT-proBNP remains the robust predictor followed by GDF-15 and ST2.

GDF-15 reflects key processes like inflammation and cardiac remodeling in HF.^6,7^ Previous studies have shown the prognostic value of baseline GDF-15 level for HF outcome.^8^ Studies that have analyzed elevated GDF-15 specifically in patients with acute HF are limited in number.^10,18–20^ Unlike a single timepoint-based measurement, repeated measurements take into account the temporal evolution as a result of the dynamic natural disease progression. Our study underscores the usefulness of repeated measurements GDF-15, which provided a better risk discrimination than a single (baseline) measurement alone. This is in accordance with the limited number of studies.^10–12^ Fluschnik et al^11^ demonstrated only a slight improvement with repeated measurements compared with our study, but there was a large interval between measurements, which could explain this discrepancy. A more frequent sampling schedule seems to be required to detect changes in biomarker level and assess risk adequately. As such, the temporal pattern revealed average GDF-15 level increased in the weeks leading up to the PE, whereas it stabilized in patients who were event-free. A similar trend was observed for NT-proBNP,^4^ ST2,^4^ and galectin-3^5^ in previous analyses of TRIUMPH. In the current study, serially measured troponin I was significantly associated with the PE but not baseline measurement, further supporting this notion.

Both baseline and repeated measurements of GDF-15 have been shown to have incremental prognostic value over NT-proBNP, the golden standard biomarker in HF.^10,12,18,20–22^ Likewise, in our study, repeated measurements of GDF-15 were associated with the outcomes independent of repeated measurements of NT-proBNP. This further denotes several, different underlying pathophysiological pathways contribute to HF progression and suggests that GDF-15 as a marker of inflammation^6^ provides additional information compared with NT-proBNP, which reflects volume overload and myocardial stretch.^23,24^ To properly assess the incremental prognostic value of serially measured GDF-15 and more accurately predict HF risk, a multimarker approach with additional biomarkers is necessary. An analysis of 14 serum biomarkers in the Bio-SHiFT study in patients with chronic HF showed a strong association of repeated measurements of GDF-15, NT-proBNP, and ST2 with the composite end point of CV mortality, heart transplantation, left ventricular assisted device and HF hospitalization.^9^ However, these associations were only analyzed separately in a clinical model and a biomarker-adjusted only model as opposed to our study where we also combined both into 1 single model. In the multimarker analysis of 7 circulating makers in the study performed by Demissei et al,^10^ in patients with acute HF, the combination of GDF-15, NT-proBNP, soluble ST2, and Hs-TnT (high-sensitive troponin T) provided significant and independent prognostic information on cardiovascular mortality. Our results show that the combination of GDF-15, NT-proBNP, and troponin I provided a favorable risk discrimination for the end points, further emphasizing the utility of joint analysis of multiple biomarkers to capture several different underlying pathways. GDF-15 was not significant in model 4 with ST2 included, possibly due to correlation or synergistic pathways and the lower area under the curve, especially on the long term, indicates more ST2 measurements are needed as the event nears to properly assess risk.

Notably, GDF-15 was more strongly associated with the mortality end point than with the PE that also includes HF hospitalization, which is in line with previous literature.^25^ This phenomenon was also observed for other biomarkers, especially troponin I, which is a marker of cardiomyocyte injury or necrosis.^26^ While it is routinely used in the diagnosis of acute coronary syndrome, previous studies have also shown the prognostic value of (isoforms of) this marker in HF.^27,28^ A study of 238 patients with advanced HF even found a relative risk of 2 for mortality after adjustment for clinical factors and BNP.^29^ Our observations confirm this independent prognostic utility of serially measured troponin I. It appears that elevated levels could possibly provide insight into the severity and etiology of acute decompensation.

Galectin-3 is another marker of systemic inflammation and fibrosis^30^ and was significantly and independently associated with adverse outcome in previous studies.^5,31^ However, in our study, its incremental prognostic value was limited when jointly modeled with other biomarkers. This might be due to its more systemic and less cardio-specific nature.

Increases in creatinine, a measure of kidney function, were associated with higher 30-day mortality or HF hospitalization in patients admitted with acute HF.^32^ However, creatinine also had limited prognostic value in our study after extensive adjustment. Nevertheless, creatinine remains important considering the close relation with HF and as a confounder due to the influence of renal clearance on biomarker levels.

HF remains a complex disease but despite GDF-15 being a pleiotropic protein involved in several pathological conditions,^34^ it enables us to elucidate the disease status and impact on cardiac functioning. Noteworthy to mention is that GDF-15 has a lower intraindividual biological variation compared with NT-proBNP,^35,36^ which is even lower in ST2, Hs-TnT, and galectin-3.^37^ Therefore, the combination of biomarkers would more reliably predict risk on an individual patient-level than relying on a single marker.

### Clinical Implications and Future Research

Based on our findings, a combination of multiple, serially measured biomarkers could play a role in risk stratification in clinical practice to discriminate between patients at low or high risk for adverse outcomes. This individualized risk assessment could be performed with a mobile/online calculator app much like the Barcelona Bio-Heart Failure risk calculator to provide up-to-date risk scores based on repeated biomarker levels and clinical confounders, hereby facilitating clinical decision-making. Furthermore, they could also prove useful in monitoring of patients as medical therapy seems to lower the levels of GDF-15 as shown in the RELAX-AHF trial (Relaxin in Acute HF), a double-blinded randomized controlled trial where patients received serelaxin versus placebo.^19^ In the CardioMEMS Heart Sensor Allows Monitoring of Pressure to Improve Outcomes in New York Heart Association Class III HF Patients trial (CHAMPION), CardioMEMS sensor invasively measured and successfully identified HF patients with elevated pulmonary pressures up to 2 weeks before decompensation allowing the physician to uptitrate medication to prevent clinical worsening and subsequent hospitalization.^38^ In our study, the longitudinal evolution of GDF-15 revealed a similar unique window to potentially, noninvasively anticipate adverse events and intervene accordingly. More research into biomarker level-guided treatment of HF is therefore warranted.^39^

### Strengths and Limitations

In this large, prospective, cohort study, specifically designed for the purpose of studying clinically relevant biomarkers in patients with acute HF, patients underwent a protocolized high-frequency (7) blood sampling during 1-year follow-up. Furthermore, a comprehensive overview is given with the analysis of both single and repeated measurements of multiple biomarkers further underscoring the merit of sequentially measuring a combination of biomarkers. Also, state-of-the-art statistical methods are applied to study the complex data that were generated by these measurements in relation to the incidence of clinically relevant end points.

Still, several limitations should also be acknowledged. First, TRIUMPH was an observational study in which the treating physician was recommended to provide HF management according to the prevailing guidelines^13^; however, adherence to these guidelines was not explicitly checked. Guideline-directed medical therapy has also been updated since (including quadruple therapy)^40^ and could therefore also affect our results and generalizability of our findings to a contemporary cohort. Furthermore, while the observational nature of the study (no stringent exclusion criteria) allowed a wide range of consecutive patients to be included, the cohort might not be fully representative of the HF population at large, for example, 37% of the patients were women. Although, despite this difference in sex distribution, we did not observe an important relation of sex on the association between the biomarkers and outcomes. Finally, while the large number of events and measurements available enabled us to run various multimarker models with adjustment for a multitude of potential confounders, we were ultimately limited by model performance (convergence) and therefore we cannot exclude the possibility of residual confounding.

### Conclusions

This multimarker analysis of the TRIUMPH study shows that repeated measurements of GDF-15 are associated with adverse outcomes in patients with acute HF, independent of several other biomarkers including NT-proBNP, which remained the most robust predictor. The multimarker model combining GDF-15, NT-proBNP, and troponin I provided a favorable risk discrimination for the end points. Our findings underscore the usefulness of both repeated measurements and a multimarker panel for improved individual patient-level prognostication. Additional studies are warranted to evaluate if these biomarkers can be (jointly) used for patient-tailored guided therapy.

## Article Information

### Acknowledgments

The authors thank all participating centers for carrying out the study procedures and data collection. All authors have read and approved the final version of the article.

### Sources of Funding

This work was supported within the framework of the Center for Translational Molecular Medicine, project TRIUMPH (grant 01C-103).

### Disclosures

Dr Asselbergs is supported by University College London Hospitals NIH and Care Research Biomedical Research Center. Dr Orsel is an employee of Philips Healthcare. Dr Pinto serves as a consultant and speaker for biotechnology and pharmaceutical companies that develop molecules that target myocardial disease, including Pfizer, Roche, Novartis, and Forbion. Dr Pinto is named inventor on patents related to cardiomyopathy therapy and holds minor shares (<5%) and spin-off aimed at developing therapies for heart disease. All other authors have nothing to disclose.
